# Isolation and Characterization of Encephalomyocarditis Virus from Dogs in China

**DOI:** 10.1038/s41598-017-00435-x

**Published:** 2017-03-27

**Authors:** Ya-Kun Luo, Lin Liang, Qing-Hai Tang, Ling Zhou, Li-Jun Shi, Ying-Ying Cong, Wen-Cheng Lin, Shang-Jin Cui

**Affiliations:** 1 Institute of Animal Science, Chinese Academy of Agricultural Sciences, Beijing, 100193 China; 2 Beijing Observation Station for Veterinary Drug and Veterinary Biotechnology, Ministry of Agriculture, Beijing, 100193 China; 3 Center for Nanyang Veterinary Biological Engineering Technology, Nanyang Normal University, Nanyang, 473061 China; 4 Department of Cell Biology, University Medical Center Groningen, 9713 AV Groningen, The Netherlands; 5 College of Animal Science, South China Agricultural University, Guangzhou, 510642 China

## Abstract

Encephalomyocarditis virus (EMCV) is as a potential zoonotic agent with a wide host range. Here, we describe an EMC virus isolate, identified as EMCV C15, which was successfully obtained from the serum of dogs from animal hospitals. Virus production in cell culture was confirmed by EMCV-specific real-time RT-PCR, indirect immunofluorescence assays and electron microscopy. In addition, the open reading frame sequence (ORF) of the EMCV C15 virus was determined. From sequence comparison and phylogenetic analysis among 24 reference EMCV strains, it appears that the EMCV C15 strain is closely genetically related to strain BEL2887A/91 (>99.0% nucleotide identity). In artificially challenged dogs, the heart and brain were important targets of EMCV C15. This study provides genetic and pathogenic characterization of the EMCV C15 strain isolated in Beijing and calls for sustained surveillance of EMCV infection in China to support better prevention and control of the disease.

## Introduction

Encephalomyocarditis virus (EMCV) belongs to the *Cardiovirus* genus of the *Picornaviridae* family^1^. *Picornaviridae* is one of the largest and most diverse families of RNA viruses and includes etiological agents that are responsible for a wide variety of human and animal diseases. EMCV is a potential zoonotic pathogen causing myocarditis, encephalitis, neurological diseases, reproductive disorders and diabetes^2^. EMCV was first isolated from a gibbon in 1945 in Florida^3^. From 1945 to the present, EMCV has been detected in many wild and domestic animals in many areas around the world, including Europe^4^, Canada^5^, South America^6^, Australia^7^, Korea^8^, Italy^9^ and China^10^. EMCV has a wide spectrum of hosts, including voles, squirrels, elephants, swine, wild boar, raccoons, antelope, lions and birds^1, 9^. In addition to its importance in animal husbandry, EMCV also has public health significance. Until 2009, no EMCV infections of humans associated with clinical signs had been reported. Nevertheless, serological studies showed that humans are susceptible to EMCV infection. For example, neutralizing antibodies against EMCV were found in 17 soldiers who presented febrile illness in the Philippines^11^. In Austria^12^, more than 5% of persons with occupational exposure to animals were EMCV sero-positive, and this percentage reached 15% for hunters. Recently, a study describing the etiology of acute febrile disease in locations across South America concluded that there is evidence supporting a role for EMCV in human infection and febrile illness^6, 13^. A new study showed that the sero-prevalence of EMCV in healthy Chinese people is approximately 30.56% (1010/3305)^14^.

EMCV is a small non-enveloped virus with a positive single-stranded genome, approximately 7.8 kb in length with a large open reading frame (ORF). The ORF codes for a polyprotein that comprises both non-structural and structural elements divided into three primary precursor molecules—P1, P2 and P3—encoding for 11 distinct proteins^15^. The function of the EMCV proteins has often been assigned by virtue of their similarity to their well-studied counterparts poliovirus (PV), Theiler’s murine encephalomyelitis virus (TMEV) and foot and mouth disease virus (FMDV).

As the presence of companion animals (especially pet dogs) becomes increasingly ubiquitous in human life, the influence of the health of such animals on human health is growing. This study describes the isolation, identification and characterization of an EMC virus, EMCV C15, from dogs. The ORF sequences of the EMCV C15 isolate were compared with the ORF of 24 EMCV strains. Phylogenetic analysis showed that the EMCV C15 strain is closely genetically related to strain BEL2887A/91 (>99.0% nucleotide identity). In dogs artificially challenged with EMCV C15, the heart and brain were important targets for the virus and exhibited a viral load of over 10^5^ gene copies. This study characterizes the molecular evolution of EMCV C15 in China and provides a reference for future studies on EMCV control and prevention.

## Materials and Methods

### Ethics Statement

All animal procedures were approved by the Animal Care Committee of the College of Animal Science, South China Agricultural University, Guangzhou, China (approval ID: 201004152). The animal experiments were conducted according to the Guide for the Care and Use of Laboratory Animals of South China Agricultural University, Guangzhou, China.

### Cell culture

Baby hamster kidney 21 (BHK21) cells were obtained from the Department of Veterinary Medicine at the Institute of Animal Science at the Chinese Academy of Agricultural Sciences. BHK21 cells were maintained in Dulbecco’s Modified Eagle’s Medium (DMEM) supplemented with 10% fetal bovine serum (FBS) (Invitrogen, USA) and 1.0 IU/mL of penicillin and streptomycin. Cells were cultured at 37 °C in an incubator containing 5% CO_2_.

### Real time RT-PCR for EMCV detection

Real-time RT-PCR assay utilized the SYBR Premix Ex TaqTM kit (Takara, Dalian, China) in a total volume of 25 μL. The assay was performed following the manufacturer’s instructions. The EMCV-specific primer set described by Wang *et al*.^16^ was used as follows: forward primer (position 1116–1140) 5′-GACGCTTGAAGACGTTGTCTTCTTA-3′; reverse primer (position 1302–1326) 5′-CCCTACCTCACGGAATGGGGCAAAG-3′. The primers were designed based on a 3D sequence and have been confirmed to be highly specific for EMCV. Real-time RT-PCR was conducted on an ABI H7900 Fast instrument (Life Technologies, Carlsbad, CA) and the results were analyzed with the included system software.

### Clinical Samples

A total of 69 serum specimens were collected from dogs at pet hospitals in Beijing in 2015. The dogs presented high fever symptoms (69/69), diarrhea (45/69) and dyspnea (32/69). Colloidal gold strip detection was used to detect canine parvovirus (CPV) and canine distemper virus (CDV). The detection results showed that 44 and 36 of 69 dogs were infected with CPV and CDV, respectively, while 22 of 69 dogs were co-infected with CPV and CDV. The results of real-time RT-PCR showed that 4 of 69 dogs were co-infected with CPV and EMCV.

### Virus isolation, propagation, and titration

The EMCV-positive serum samples were incubated with 1.0 IU/mL penicillin/streptomycin for 10 min at 37 °C. Then, serum samples were filtered through a 0.22-μm filter and used as an inoculum for EMC virus isolation. Isolation of EMCV C15 was attempted using BHK21 cells as previously described with some modifications^17^. Confluent BHK21 cells in 6-well plates were washed twice with postinoculation medium and inoculated with 300 μL of sample and 100 μL of postinoculation medium. After 45 min, another 1.0 mL of DMEM supplemented with 10% FBS and 1.0 IU/mL of penicillin and streptomycin was added to each well in the 6-well plate. Inoculated cell (passages 0 [P 0]) were incubated at 37 °C with 5% CO_2_. When a 70% cytopathic effect (CPE) developed, the plates were subjected once to freezing and thawing. The mixtures were centrifuged at 3,000× *g* for 15 min at 4 °C. The supernatants were harvested for further propagation or saved at −80 °C. If no CPE was observed three days post-inoculation, the plates were frozen and thawed once, after which the supernatants were inoculated on new BHK21 cells for a second passage. Inoculated cells at each passage were also tested using a real-time RT-PCR assay. If the CPE tests and real-time RT-PCR results were negative after four passages, the virus isolation result was considered negative. Virus titration was performed in 96-well plates with 10-fold serial dilutions performed in triplicate per dilution. Virus titers were determined according to the Reed and Muench method and expressed as the 50% tissue culture infective dose (TCID_50_)/100 μL. The virus isolated and characterized in this study was designated EMCV C15.

### Electron Microscopy (EM)

Samples were prepared for negative staining examination by electron microscopy (EM) following previously described procedures with some modifications^18^. BHK21 cells infected with the EMCV-C15 P3 virus isolate were frozen and thawed 48 h post-infection, after which they were centrifuged at 3,000× *g* for 15 min. A total of 5.0 mL of supernatant was prepared in this section. The virus was pelleted from the supernatant by ultracentrifugation at 159,000× g for 1.5 h at 4 °C and resuspended in PBS. The resulting pellet was resuspended in 500 μL of 0.01 M PBS (pH 7.2 to 7.4), which was nebulized onto coated EM grids. The grids were stained with 1% phosphotungstic acid (pH 7.0) and observed with a transmission electron microscope.

### Indirect immunofluorescence assay (IFA)

BHK-21 cells were infected with EMCV-C15 (m.o.i. of 1.0). At 48 h post-infection, the infected BHK-21 cells were fixed with 4% formaldehyde for 30 min at room temperature. The cells were washed three times with PBS, blocked with 10% FBS for 1 h at room temperature, washed three more times with PBS and incubated with mouse anti-VP1 mAb^19^ (1:200 dilution) for 1 h. The cells were washed three times with PBS and incubated with FITC-conjugated goat anti-mouse IgG or TRITC-conjugated goat anti-mouse IgG (1:100 dilution) for 1 h at 37 °C. After three more washes in PBS, the cells were imaged under an inverted fluorescence microscope (EVOS f1, AMG, USA).

### Western blot analysis

BHK-21 cells were infected with EMCV-C15 (m.o.i. of 1.0) and with EMCV HB10 as a positive control. At 48 h post-infection, cells were lysed in 5× SDS-PAGE loading buffer. The protein concentrations were determined with a bicinchoninic acid protein assay reagent (Pierce) according to the manufacturer’s instructions. An equal amount of each sample was loaded onto a 12% SDS-PAGE gel, followed by protein transfer to a PVDF membrane (Millipore). Each membrane was blocked for 1 h with blocking buffer (5% skimmed milk and 0.1% Tween-20 in PBS) and incubated with anti-VP1 primary antibody (1:1,000 dilution)^19^ or with mouse anti-GAPDH antibodies (Cell Signaling Technology, Inc.) to detect endogenous GAPDH, which served as a protein-loading control. Each membrane was washed three times with washing buffer (0.1% Tween-20 in PBS) and incubated with horseradish peroxidase-conjugated secondary antibodies (1:2000 dilution) for 1 h, after which the membranes were washed and exposed to the detection agent 3,3′-N-diaminobenzidine tertrahydrochloride (DAB, CWBIO, China). Protein sizes were determined by comparison with prestained protein ladders (Thermo Scientific, U.S.A.).

### Experimental infection of mice

Thirty six-week-old specific-pathogen-free (SPF) BALB/c mice were randomly divided into six groups of five mice each. Groups 1–5 were each inoculated intraperitoneally (i. p.) with 100 μL of different doses (10^5^, 10^4^, 10^3^, 10^2^, or 10^1^ TCID_50_) of EMCV-C15, and mice in group 6 were injected with 100 μL of DMEM (uninfected control group). Following inoculation, clinical signs were monitored daily. The median lethal dose (LD_50_) was performed as previously described^20^.

### Animal experiments and detection of viral load in dogs

Eight 25-to-30-day-old dogs were obtained from a commercial breeding herd that was free of CPV, CDV, CPIV, CHV, ICHV and EMCV. The dogs were observed for two weeks to ensure that none were symptomatic and were then randomly assigned to two groups: group A (n = 5) and group B (n = 3). Each dog in group A was treated with 1.0 mL (10^5^ TCID_50_) of EMCV-C15, whereas each dog in group B was treated with 1.0 mL of DMEM. At 35 DPI, the animals in group A and group B were euthanized and the viral load in several organs (heart, liver, spleen, lung, kidney and brain) was determined by real-time RT-PCR performed as described above. Each sample was tested three times.

### Sanger sequencing and phylogenetic analysis

EMCV-specific primers (Table 1) were designed based on the ORF of the BJC3 strain (DQ464062). DNA fragments corresponding to the complete ORFs of EMCV C15 strain were amplified and the amplicons were sequenced commercially (HuaDa, Beijing). Sequence assembly was carried out using the SeqMan program included with the DNASTAR software package (Madison, WI). The complete nucleotide sequence of the EMCV-C15 ORF was deposited in GenBank under accession number KU664327. Phylogenetic analysis was performed using the nucleotide sequence of the EMCV-C15 virus from this study, as well as the sequences of 24 reference strains for which full genome sequences were available in GenBank. A phylogenetic tree was constructed based on the ORF sequences of EMCV-C15 using the distance-based neighbor-joining method in the MEGA 5.1 software package. Bootstrap analysis was carried out on 1,000 replicate data sets.

**Table 1:** Primers used for RT-PCR amplification.

Primer name	Primer sequence (5′–3′)	Position*	Amplified fragment (bp)
EMCV-P1	CTGTCTTCTTGACGAGCATTC	285–305	944
EMCV-P2	CCACTGTTGACTGGGTGTTT	1209–1229	
EMCV-P3	GGAAGGCAATGAAGGTGTG	967–986	890
EMCV-P4	GGCTGAATAGAAGCGGTGA	1568–1587	
EMCV-P5	TGGAAGTTTGCTGGTGTTC	1507–1526	1001
EMCV-P6	CATCCTGGTGGGTAAGTGAG	2488–2508	
EMCV-P7	TTCACTGGGACYGCGATGATGAA	1948–1971	1701
EMCV-P7	TCGGCAGTAGGGTTTGAG	3631–3649	
EMCV-P7	GGTTTGGAGGTTAGATTGTTTAG	3500–3523	1072
EMCV-P8	TTCTCTTTCACTGCCTGATTGT	4550–4572	
EMCV-P9	AGGTGGTTGATTGGTTTGG	4383–4402	998
EMCV-P10	CTGTCGCTTCCTGTCTTGTT	5361–5381	
EMCV-P11	TCCCTGGTAGATGTGATTGAG	5198–5219	671
EMCV-P12	AGAGAGGCGGATGAAAGATA	5849–5869	
EMCV-P13	TTGTTGGACATTCAGGGAC	5594–5613	864
EMCV-P14	CTCTATTGGCATACTCTTTGG	6437–6458	
EMCV-P15	AGAGGCTGATGTAGATGAAGTG	6358–6380	696
EMCV-P16	GGTTGAAATGGCTAATGACT	7034–7054	
EMCV-P17	GTTCAAGCCGCCAAGACA	6725–6743	728
EMCV-P18	CCCTGGACGATAGTATGACAAC	7431–7453	
EMCV-P19	ATTAGCCATTTCAACCCA	7039–7057	655
EMCV-P20	GCAAAGTAGAAGAACCCTGT	7674–7694	

EMCV-specific primers were designed based on the ORF sequence of the BJC3 strain (DQ464062). DNA fragments corresponding to the ORF of EMCV-C15 were amplified, and amplicons were sequenced commercially (Huada, Beijing). Sequence assembly was carried out using the SeqMan program included with the DNASTAR software package (Madison, WI).

*Nucleotide position is based on the sequence of EMCV strain BJC3 (DQ464062).

## Results

### Virus isolation and characterization

**Figure 1:**
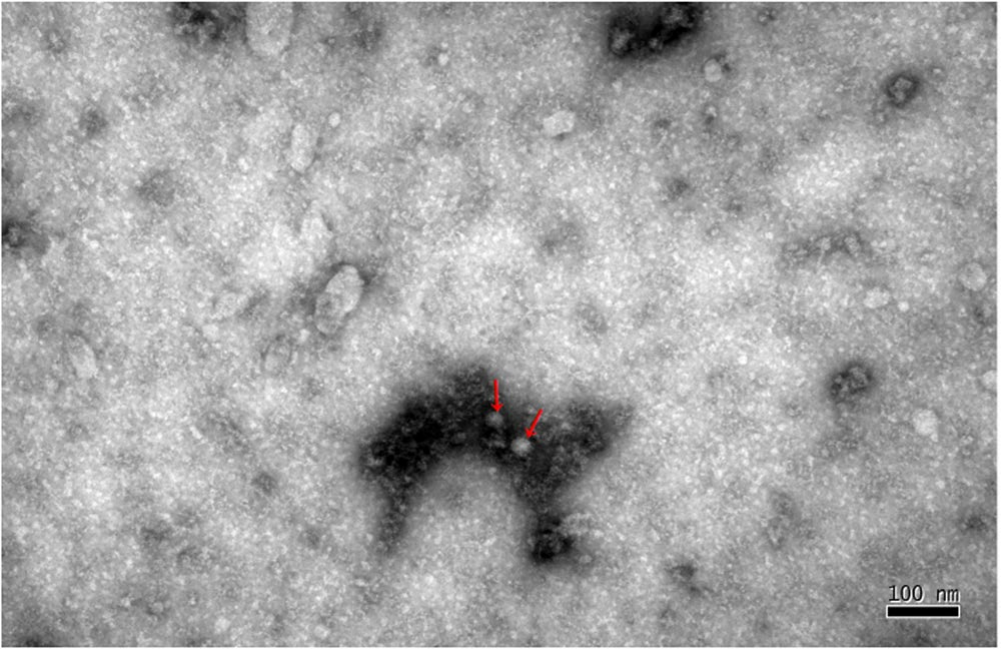
Electron micrographs showing the EMCV-C15 isolate. Virus was concentrated from the tissue culture fluid of virus-infected BHK21 cells. 50,000× magnification. Scale bar represents 100 nm.

**Figure 2:**
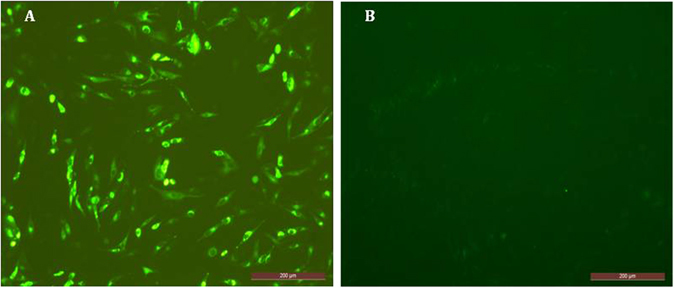
Indirect immunofluorescence assay (IFA). EMCV-C15-infected BHK21 cells and uninfected control cells were examined by IFA using EMCV-specific monoclonal antibodies ((**A**), EMCV C15-infected BHK21 cells; (**B**), negative control BHK21 cells; scale bar, 200 μm).

**Figure 3:**
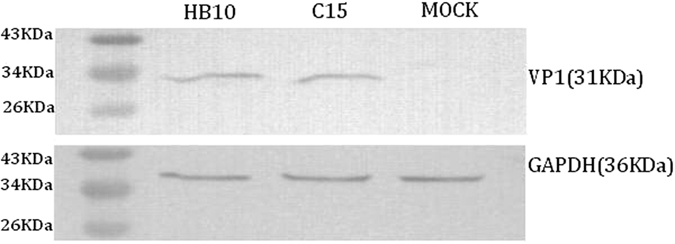
Western blot analysis. BHK21 cells were infected at an MOI of 1.0 for 48 h. The total cell extracts from uninfected and infected cells were analyzed by western blotting using a mouse anti-EMCV VP1 monoclonal antibody. The same blots were reprobed with mouse anti-GAPDH antibody. Molecular size markers are indicated to the left of each blot.

An EMCV isolate designated EMCV-C15 was successfully obtained from a mixture of four serum samples from pet hospitals in Beijing (collected in March 2015) which had shown positive real-time RT-PCR results. The EMCV-C15 particles in infected BHK21 cells were examined by EM 48 h post-infection. As shown in Fig. 1, negatively stained samples contained a small virus with a diameter of 27–30 nm^18^. Virus growth was confirmed by IFA using an anti-EMCV VP1 specific monoclonal antibody^19^. The EMCV VP1 protein stained by the monoclonal antibody was distributed in the cytoplasm but not in the nucleus (Fig. 2A). Negative control cells are shown in Fig. 2B. Western blot analysis demonstrated that capsid protein VP1 expression was similar in BHK-21 cells infected with EMCV-C15 or EMCV HB10, which was used as a positive control (Fig. 3). Taken together, these results show that EMCV-C15 and EMCV HB10 are indistinguishable with regard to viral replication and spreading in BHK-21 cells and that the monoclonal antibody recognizes an epitope common to both strains.

### EMCV-C15 infection in artificially challenged mice

Mice injected with EMCV-C15 showed clinical symptoms including anorexia, paralysis and sudden death, and displayed high mortality. At 15 DPI, the mice that were still alive were bled and subjected to pathological examination. The presence of EMCV-C15 was confirmed by real-time RT-PCR. The LD_50_ of EMCV-C15 in mice was 10^2.98^ TCID_50_ (Table 2).

**Table 2:** Outcome of infection with EMCV C15 in mice.

Group	Doses (TCID50)	Amounts	Mortality	LD50 (TCID50)
EMCV C15	10^5^	5	5/5	10^2.98a^
	10^4^	5	4/5	
	10^3^	5	2/5	
	10^2^	5	2/5	
	10^1^	5	0/5	
DMEM	0.1 mL	5	0/5	

^a^Data were analyzed using SPSS 17.0 software. One-way ANOVA followed by Duncan’s multiple range test was used to compare the parameters among the different groups.

### Quantity and distribution of EMCV-C15 in artificially challenged dogs

**Figure 4:**
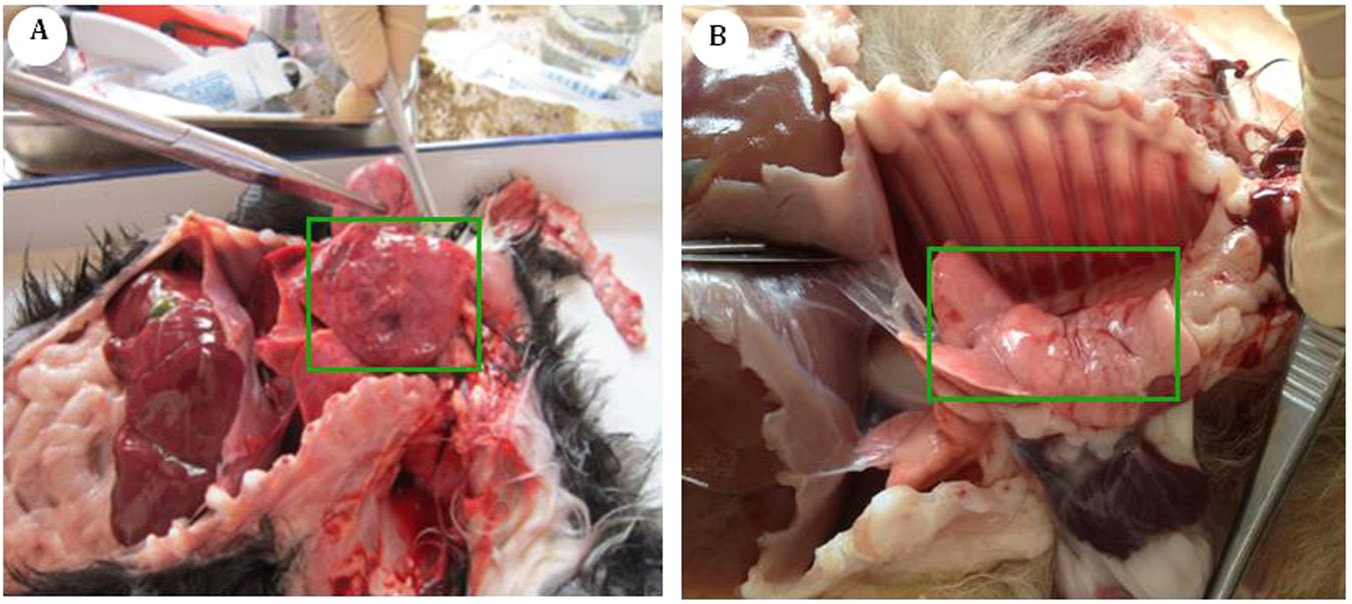
Experimental infection of dogs to assess the distribution of EMCV-C15 in various organs. Dogs in group A were challenged with EMCV-C15 and presented with pulmonary edema (**A**). The control dogs in group B, which were not inoculated with the virus, displayed no clinical signs (**B**).

**Figure 5:**
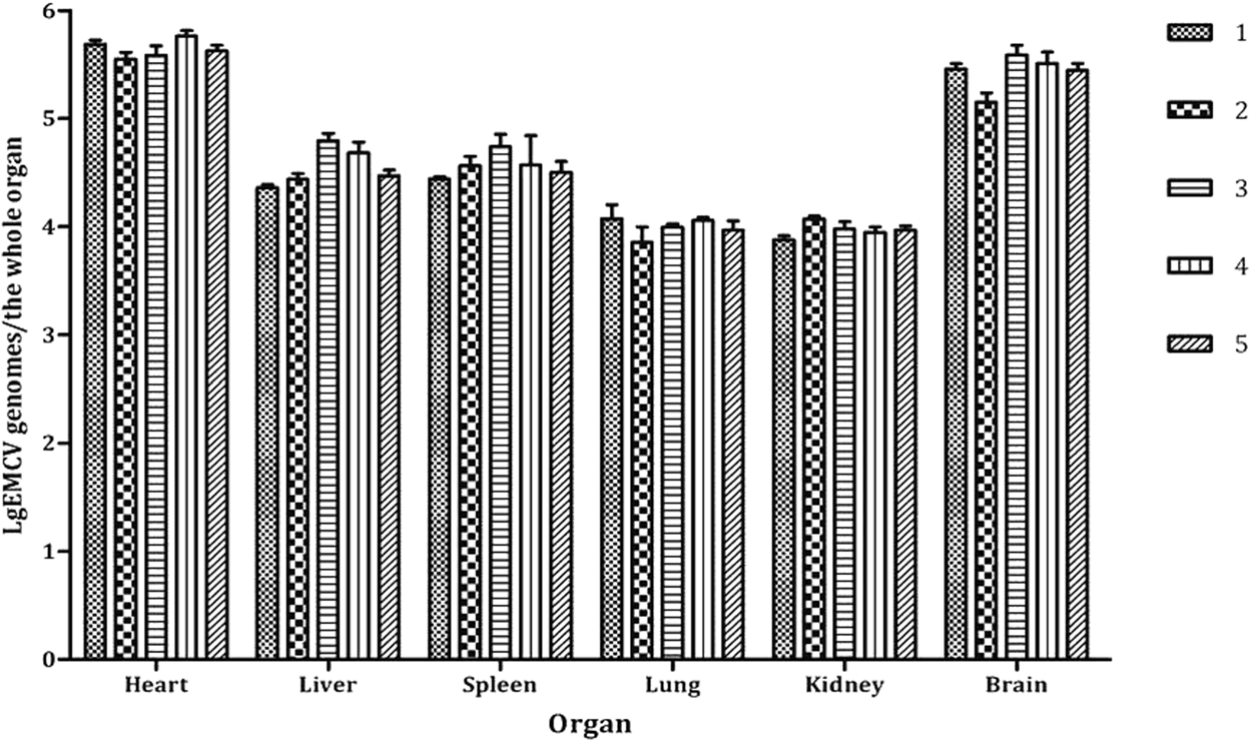
EMCV-C15 loads in the organs of artificially challenged dogs according to real-time RT-PCR. Viral loads in various organs are shown in the graph. Eight 25-to-30-day-old dogs were obtained from a commercial breeding herd that was free of CPV, CDV, CPIV, CHV, ICHV and EMCV. Dogs were randomly allocated into two groups. Dogs in group A (n = 5) were inoculated with 1.0 mL (10^5^ TCID_50_) of EMCV-C15. Dogs in group B (n = 3) were injected with 1.0 mL of DMEM as a control treatment. At 35 DPI, all dogs were euthanized and subjected to analysis of viral loads/virus distribution of EMCV-C15. Numbers 1, 2, 3, 4 and 5 in the legend correspond to the five dogs inoculated with EMCV-C15. Data from group B (uninfected group) are not visible in the graph because there was no viral load in animals from that group. Error bars represent the standard error of the mean of three replicates.

The five dogs in group A, which had been inoculated with EMCV-C15, presented with pulmonary edema (Fig. 4A), actuated pericardial effusion, myocarditis, hind limb paralysis and encephalitis (pictures not shown). However, the dogs in group B displayed no clinical signs (Fig. 4B). The presence of EMCV-C15 in the organs of each group was detected by real-time PCR. In group A, EMCV was detected in the heart, liver, spleen, lung, kidney and brain, which had average viral loads of approximately 4.37 × 10^5^, 3.52 × 10^4^, 3.64 × 10^4^, 9.72 × 10^3^, 9.27 × 10^3^, and 2.68 × 10^5^ EMCV genomes, respectively (Fig. 5).

### Sequence comparison and phylogenetic analysis of EMCV-C15

**Figure 6:**
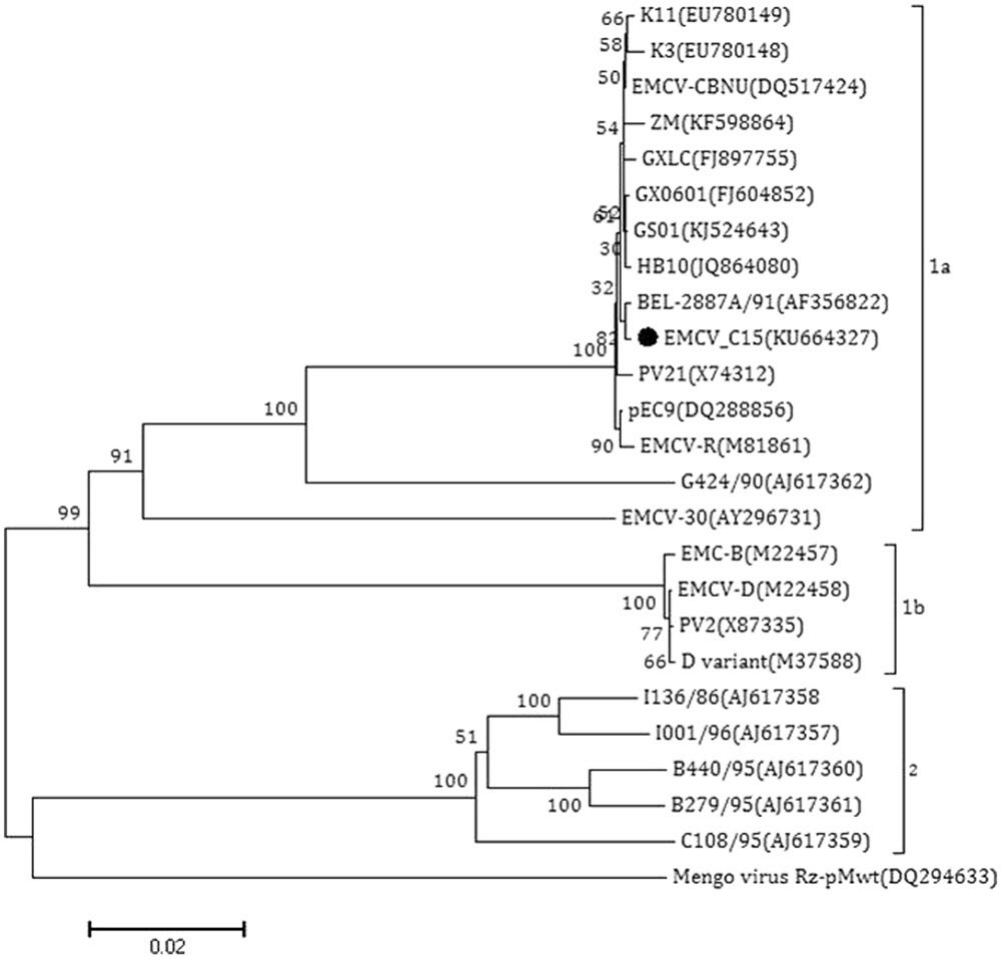
Phylogenetic analysis of isolate EMCV-C15 based on the complete ORF of 6879 nt. A phylogenetic tree was constructed using MEGA5.1 software. Mengovirus served as an outgroup. Bootstrap values obtained from 1,000 replicates are shown at the major nodes. The isolate identified in this study is indicated with solid dots.

Pairwise comparisons of the nucleotide sequences were performed to examine the degree of sequence similarity between EMCV C15 isolate and 24 EMCV reference strains retrieved from GenBank nucleotide database (Table 3). The complete 6,879-nt EMCV C15 ORF GenBank accession number (KU664327) encoding 2,292 amino acids with no insertions or deletions was examined. Multiple sequence alignment based on the ORF sequence of EMCV-C15 and other EMCV reference strains was performed using the Clustal X program^17^. The results showed the newly isolated virus (EMCV C15) shared the highest sequence similarity (99.0–99.75%) with GX0601, BJC3, NJ08, GS01, HB10 and BEL-2887A/91. However, it shared lower similarity (80–83.52%) with PV2, EMCV-B, EMCV-D, and the D variant. Phylogenetic analyses of the ORF sequence of the EMCV C15 in this study demonstrated that all EMCV strains could be divided into two mian groups (groups 1 and 2). Group 1 was subdivided into group 1a and b (Fig. 6). Except EMCV-R and EMCV C15 strains, the rest of strains were isloated form pigs in group 1a. The EMCV-C15 strain was clearly most closely related to the EMCV strain BEL2887A/91 which was detected in 1991 in Belgium. Group 2 comprises five EMCV isolates from *sus scrofa*.

**Table 3:** Encephalomyocarditis virus isolates used in this study.

Viral designation	Accession no.	Geographic origin	Host species	Nucleotides (bp)	Isolation year
EMCV30	AY296731	USA	Pig	7678	1987
CBNU	DQ517424	South Korea	Pig	7713	2006
GX0601	FJ604852	China	Pig	7729	2006
GXLC	FJ897755	China	Pig	7725	2009
HB10	JQ864080	China	Pig	6879	2012
ZM	KF598864	China	Pig	6861	2013
GS01	KJ524643	China	Pig	7717	2014
K11	EU780149	South Korea	Pig	7744	1990
PV2	X87335	Germany	Pig	7820	1985
EMCV-B	M22457	USA	Pig	7825	1980
EMCV-D	M22458	USA	Pig	7829	1980
D variant	M37588	Panama	Pig	7842	1980
PV21	X74312	Germany	Pig	7861	1993
BEL-2887A/91	AF356822	Belgium	Pig	7730	1991
pEC9	DQ288856	USA	Pig	7722	1995
K3	EU780148	South Korea	Pig	7731	1990
EMCV-C15	KU664327	China	Dog	6879	2015
MengoRz pMwt	DQ294633	USA	Mouse	7765	1989
EMCV-R	M81861	USA	Chimpanzee	7835	1945
I001/96	AJ617357	Italy	*Sus scrofa*	2703	1996
I136/86	AJ617358	Italy	*Sus scrofa*	2703	1986
C108/95	AJ617359	Cyprus	*Sus scrofa*	2703	1995
B440/95	AJ617360	Belgium	*Sus scrofa*	2703	1995
B279/95	AJ617361	Belgium	*Sus scrofa*	2703	1995
G424/90	AJ617362	Greece	*Sus scrofa*	2703	1990

Multiple sequence alignment based on ORFs and other strains available from the GenBank nucleotide database was completed using the Clustal X program.

## Discussion

EMCV has often been described as a potential zoonotic agent with a wide range of hosts. Reports have clearly shown that humans are susceptible to EMCV infection; indeed, such infection is likely to be fairly common, but most human cases are probably asymptomatic and/or unrecognized^21^. Although human exposure to EMCV is quite common and associated with low morbidity, the virus is a potential threat to public health security. Therefore, it is essential that researchers focus on EMCV infection in humans and dogs. Infection of dogs with EMCV was reported by Schwab *et al*., who described a high proportion of dogs and cats with non-suppurative meningoencephalitis, for which immunohistochemical examination with antibodies against 18 different infectious agents failed to reveal a cause. However, the significance of positive immunoreactions obtained with antibodies against proteins produced by West Nile virus (WNV) and EMCV requires further investigation^22^.

In this study, 4 of 69 dog serum specimens tested positive for EMCV using real-time RT-PCR. EMCV-C15 was isolated from a mixture of four serum samples that were co-infected with CPV and EMCV. The EMCV-C15 isolate was serially propagated via cell culture methods and characterized. By examining CPE development, IFA staining, EM, western blot analysis, infectious virus titers and complete ORF sequences, we clearly demonstrated that the C15 isolate is phenotypically and genetically stable in cell culture.

Experiments in mice showed that the EMCV isolate was highly virulent and caused hind limb paralysis. In addition, we revealed that the LD_50_ of EMCV-C15 in mice was 10^2.98^ TCID_50_. To enhance our understanding of EMCV loads in various organs in dogs, real-time RT-PCR was used to quantify the presence of the EMCV genome in various tissues from artificially challenged dogs. The heart and brain are the most important targets for the isolated EMCV C15 in challenged dogs. Inflammation of the heart and brain were obvious in dogs infected with EMCV-C15 and might have been important in the development of acute myocarditis and pulmonary edema.

Phylogenetic analysis showed that the C15 strain is closely genetically related to strain BEL2887A/91, which circulated in Belgium in 2002. The hosts of BEL2887A/91 are pigs or piglets; however, the mechanism by which EMCV was introduced to dogs remains unknown. The history of the spread of EMCV among species and geographical areas merits further investigation.

In this study, the EMCV isolate EMCV-C15 was obtained and characterized. To our knowledge, this is the first report describing the isolation and characterization of EMCV from dogs. All experimental data indicate that the dog is a host for EMCV. Therefore, people living in areas with dogs, especially owners of pet dogs, should be aware of this potential health threat.
